# Spectator Exciton Effects in Nanocrystals III: Unveiling the Stimulated Emission Cross Section in Quantum Confined CsPbBr_3_ Nanocrystals

**DOI:** 10.1021/jacs.4c05412

**Published:** 2024-07-15

**Authors:** Apurba De, Soumyadip Bhunia, Yichao Cai, Tal Binyamin, Lioz Etgar, Sanford Ruhman

**Affiliations:** ‡ Institute of Chemistry, The Hebrew University of Jerusalem, Jerusalem-91904, Israel

## Abstract

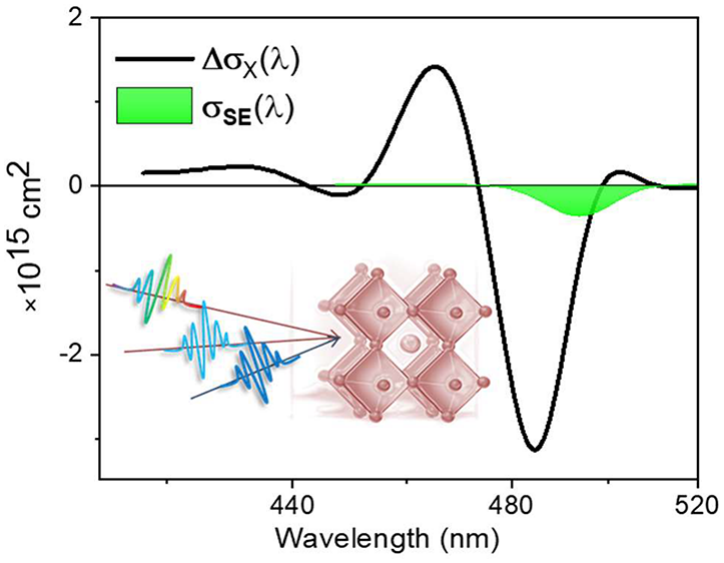

Quantifying stimulated emission in semiconductor nanocrystals (NCs) remains challenging due to masking of its effects on pump–probe spectra by excited state absorption and ground state bleaching signals. The absence of this defining photophysical parameter in turn impedes assignment of band edge electronic structure in many of these important fluorophores. Here we employ a generally applicable 3-pulse ultrafast spectroscopic method coined the “Spectator Exciton” (SX) approach to measure stimulated-emission efficiency in quantum confined inorganic perovskite CsPbBr_3_ NCs, the band edge electronic structure of which is the subject of lively ongoing debate. Our results show that in 5–6 nm CsPbBr_3_ NCs, a single exciton bleaches more than half of the intense band edge absorption band, while the cross section for stimulated emission from the same state is nearly 6 times weaker. Discussion of these findings in light of several recent electronic structure models for this material proves them unable to simultaneously explain both measures, proving the importance of this new input to resolving this debate. Along with femtosecond time-resolved photoluminescence measurements on the same sample, SX results also verify that biexciton interaction energy is intensely attractive with a magnitude of ∼80 meV. In light of this observation, our previous suggestion that biexciton interaction is repulsive is reassigned to hot phonon induced slowdown of carrier relaxation leading to direct Auger recombination from an excited state. The mechanism behind the extreme slowing of carrier cooling after several stages of exciton recombination remains to be determined.

## Introduction

Using Einstein’s equations, rates for all modes of interaction between a two-level atom and light can be predicted after characterizing any one of the three defining constants, the stimulated emission cross section (σ_SE_), the absorption coefficient, or the radiative lifetime.^1^ In contrast, despite their frequent characterization as “artificial atoms”,^2^ semiconductor nanocrystals (NCs) exhibit far more complex photophysics. Closely spaced electronic levels near the band edge (BE), the lowest of which is regularly optically inaccessible from the ground state, pose difficulties in the application of NCs as fluorophores.^3^ Near overlap of absorption and emission from the lowest excited state in nanodots of most semiconducting materials further limits their use in long-lived optical gain media.^4−6^ Along with coupling of the discrete electronic levels near the BE to phonons, these properties make for a complicated dependence of NC emission on temperature and surface chemistry.^7−9^

A study by Gong et al. has attempted to reconcile the Einstein constant approach to the photophysics of CdSe NCs. Their findings highlight specific intricacies of the NC photophysics and show that without prior information concerning state degeneracies and transition strengths, absorption and emission spectra alone leave the system underdefined.^10^ Ultrafast transient absorption (TA) experiments can provide some of the missing information. Yet, numerous such investigations have still not clarified the apparent absence of hole state-filling effects on TA spectra of CdSe NCs, by far the most extensively studied semiconductor NCs.^11−14^ One factor which limits the information obtainable from TA is the characteristic spectral overlap of ground state absorption bleach with excited state absorption and stimulated emission. The ability to separate all three contributions is key to figuring out the underlying electronic structure. Two-dimensional electronic spectroscopy (2DE) has been utilized for that purpose.^15^ However, the low amplitude of stimulated emission in lead chalcogenide NCs due to the BE level degeneracies along with the extensive analysis which is applied in 2DE complicates interpretation of the obtained spectra. Thus, while characterization of all three radiative processes in NCs would greatly help in assigning the level structure, no general method provides such delineation, particularly of the often weak cross section for stimulated emission (SE) σ_SE_.

Such a method would be particularly helpful in studying the photophysics of Lead halide perovskite (LHP) NCs where, despite immense R&D efforts spent on their photonic applications,^16^ BE level structure remains controversial. Recent advances in synthesis enable preparation of monodisperse samples sized within the regime of quantum confinement (QC).^17−19^ These samples exhibit unique photophysical properties, such as unprecedented size-dependent brightness of the lowest lying exciton state, and unusual photoinduced excited state absorption bands.^20−23^ Understanding these behaviors hinges on determining the BE electronic structure. Some experiments supported by theoretical models assign brightness of the band edge exciton to inversion of state ordering due to Rashba coupling.^20^ In contrast, recent atomistic calculations have not reproduced this effect, and in line with PL experiments show that the lowest exciton state is dark for QC particles.^23^

After pioneering preparation of monodisperse QC CsPbBr_3_ NCs,^17^ the Son group reported an unusual induced absorption feature midway between the two lowest exciton peaks in relaxed monoexcitons.^24^ This feature was assigned by Rossi et al. to a perturbative allowing of a forbidden mixed angular momentum exciton state. In a previous study conducted in our lab, pump–probe (PP) experiments on a series of QC CsPbBr_3_ samples were conducted to test this assignment.^25^ The observation that a single exciton blocks a major fraction of the lowest energy exciton absorption band, and that a second one mainly bleaches the above-mentioned induced absorption, led to our suggestion that biexciton interaction in these samples was repulsive and not attractive as is usually the case in semiconductor NCs.

A number of recent papers have challenged this assignment.^21,26,27^ Time and frequency resolved single particle photon counting photoluminescence has identified red-shifted biexciton emission indicating strong biexciton attraction.^26^ A similar conclusion was derived from two recent polarization dependent TA studies on QC CsPbBr_3_ NCs. In the latter various polarization combinations were applied to separate the stimulated emission contributions in TA for short-lived coherent excitons.^27^ They show that while this coherence persists, the single exciton can provide a net optical gain from BE stimulated emission. This approach, however, does not provide similar information for the long-lived and therefore practically more relevant optically dephased single exciton state.

In view of the perceived importance of LHP NCs as a next-generation photonics platform and continued uncertainties concerning underlying electronic structure, we have applied three pulse spectator exciton (SX) experiments to quantify σ_SE_(λ) in QC CsPbBr_3_ NCs. The results were corroborated by conducting subps time-resolved photoluminescence (TRPL) on the same particles. The spectator exciton approach compares identical PP sequences, once on unexcited NCs, and then on a sample which has been uniformly excited with one cold exciton per particle–the “spectator”.^28,29^ The latter can be generated by high intensity above BE excitation, which initially generates a distribution of multiexciton states. Rapid Auger recombination then leads to long-lived single excitons in all particles which have absorbed one or more photons. Longevity of single excitons allows cooling of the SXs before the delayed PP sequence.

By comparing TA with and without SXs we have determined σ_SE_ from the single exciton state in QC CsPbBr_3_ NCs, showing it to be roughly 6 times weaker than the accompanying band edge bleach per exciton. The unique ability to quantify σ_SE_ even in the presence of overwhelming excited state absorption is very important given its sensitivity to BE state degeneracy and selection rules for transitions among them. In addition, comparison of TRPL and SX experiments proves that exciton–exciton interactions in QC CsPbBr_3_ NCs are strongly attractive (∼100 meV). Accordingly hot biexciton cooling following several stages of multiexciton recombination is prolonged from less than 1 ps in the absence of such “preheating”, to several psec, rendering it to be slower than the Auger recombination itself.

## Experimental Section

### Synthesis of Different Sized CsPbBr_3_ NCs

#### Chemicals

Cesium carbonate (Cs_2_CO_3_, 99.9%, Sigma-Aldrich), lead(II) bromide (PbBr_2_, ≥98%, Sigma-Aldrich), zinc(II) bromide (ZnBr_2_ 99.999% m.b., Holland-Moran), oleic acid (OA, 90%, Sigma-Aldrich), oleylamine (OLAM, 70%, Sigma-Aldrich), 1-octadecene (ODE, 90%, Sigma-Aldrich), ethyl acetate (≥99.5%, Sigma-Aldrich), and hexane (not pure, Gadot) were purchased and used as received, without any further purification.

#### Preparation of Cs-Oleate

The Cs-oleate precursor was prepared according to a previously published procedure by Dong et al.^17^ In a 100 mL 3-neck flask, 0.25 g of Cs_2_CO_3_ (0.76 mmol) were mixed with 900 μL of oleic acid (OA) and 9 mL of 1-octadecene (ODE). The solution was degassed for 1 h under vacuum conditions at 120 °C and then heated to 150 °C under an argon flow.

#### Synthesis of CsPbBr_3_ NCs

The NCs were synthesized according to Dong et al.^17^ First, 0.2 mmol of PbBr_2_ was mixed with 0.7 mmol of ZnBr_2,_ 0.5 mL of OA, 0.5 mL of OLA, and 5 mL of ODE in an additional 100 mL 3-neck flask. The solution was degassed for 1 h under vacuum at 120 °C and then heated to 140–190 °C (for different sizes) under an argon flow. The reaction was carried out by injecting 0.4 mL of the Cs-oleate precursor solution into the PbBr_2_ precursor solution using a preheated syringe. The reaction was quenched by using an ice bath after a few seconds. Ethyl acetate was added to the crude solution in a volume ratio of 3:1, and the NCs were centrifuged at 6000 rpm for 10 min. The precipitate was dispersed in hexane, and the NCs dispersion was centrifuged again at 3000 rpm for 5 min to get rid of aggregates and unreacted salts.

#### High Resolution Transmission Electron Microscopy (HR-TEM)

Morphology of the NPs was analyzed with an HR TEM (High Resolution Transmission Electron Microscope) Tecnai F20 G2 (FEI Company, U.S.A.). Samples preparation was performed as follows: 2.5 μL of the NCs dispersion were dropped on an ultra-light copper grid coated with amorphous carbon film.

#### Femtosecond Pump–Probe Measurements

Two different home-built transient absorption setups were appointed for the TA measurements of different NCs, details of which can be found elsewhere.^30,31^ Briefly, for 6 nm NCs measurements, the fundamental ∼800 nm pulse of ∼35 fs pulse width and 1 mJ energy per pulse at 1 kHz repetition rate were generated by a multipass amplified Ti-sapphire laser system. To generate the pump pulse, a home-built noncollinear optical parametric amplifier (NOPA) generating tunable broadband pulses between 500 and 700 nm is used. However, in this case, a narrow band is generated and tuned accordingly. To generate a 400 nm pump pulse, a fraction of the 800 nm fundamental is frequency doubled using a BBO crystal. This is further split into two; one to generate an intense 400 nm saturation pulse (for three pulse measurements) and a weak 400 nm pulse. Probe pulses in the range of 400–750 were generated by focusing 1300 nm pulses from an optical parametric amplifier (TOPAS 800, Light Conversion) on 2 mm of CaF_2_. The probe was dispersed using a SpectraPro 2150i imaging spectrograph (Acton Research Corp.) equipped with a CCD camera (Entwicklungsbüro Stresing). While for the TA measurements of 5 nm NCs, the fundamental laser beam of 800 nm is generated using another home-built multipass amplified Ti:sapphire setup, which produces ∼30 fs, 0.8 mJ pulses at the rate of 600 Hz. The pump pulses were generated using TOPAS (light conversion), by mixing signal or idler with the fundamental and tuned accordingly. The saturation 400 nm (used for three pulse measurement) and the weak 400 nm were generated separately by frequency doubling of a fraction of the 800 nm output of the amplifier. The white light continuum probe pulses (400–700 nm) were generated by focusing the 800 nm fundamental pulses on the 2 mm CaF_2_ crystals. The Probe intensity was dispersed through an imaging spectrograph (Oriel-Newport MS260i) equipped with a CCD (Andor technologies, Newton. On the sample, the spot size of the pump pulse was at least three times larger than the probe pulse. In all measurements, the samples were placed in a 0.5 mm path length airtight quartz cell (filled in an inert atmosphere inside the glovebox) and kept under rotation to avoid photocharging or photodegradation.

##### Three Pulse Pump–Probe Measurements

In the case of three-pulse TA measurements, the two-pulse pump–probe experiments were repeated in the presence of another saturation pulse at 400 nm, which is earlier than the second weak pump. The delay between the saturation and the weak pump was set to 50 ps for this case (when Auger is over). The constant beach signal (if any) after the completion of the AR process in the raw data of the three pulse measurements is a direct measure of the unsaturated NCs. Thus, to eliminate the contribution of the unsaturated NCs in the three pulse data, the raw data from three-pulse measurements were subtracted by the same fraction of the obtained signal in the two-pulse data. Finally, the subtracted data set was normalized to the fraction corresponding to the total population of saturated NCs.

#### Time-Resolved PL Measurement

Time-resolved PL measurements were carried out on a home-built Kerr gate fluorescence setup detailed in Figure S1A.^32^ The fundamental beam of a multipass amplified Ti-sapphire laser system (30 fs, 0.8 mJ) serves to generate the pump pulse by frequency doubling, and the gate beam is polarized at 45°. Forward emerging fluorescence after 400 nm excited NCs was collimated and refocused on the Kerr medium (NSF-6, 1.5 mm) with a pair of off-axis parabolic mirrors. A pair of high contrast polarizers with orthogonal alignment were placed before and after the Kerr medium ensuring minimum emission leak under ungated conditions. The time-gated fluorescence is obtained by delaying the gate pulse. On the Kerr medium, the spot size of the fluorescence was kept comparable to that of the gate beam. Similar to the pump–probe, the NCs were placed in a 0.5 mm airtight quartz cell under rotating conditions to avoid sample degradation. The fluorescence signal is collimated using an achromatic lens and then directed to an imaging spectrograph (Oriel-Newport MS260i) equipped with a CCD (Andor technologies, Newton). The instrument response time of this setup, as measured by the Gaussian fit to the first derivative of the integrated PL rise of DCM dye fluorescence, is found to be ∼0.4 ps (Figure S1B).

## Results

**Figure 1:**
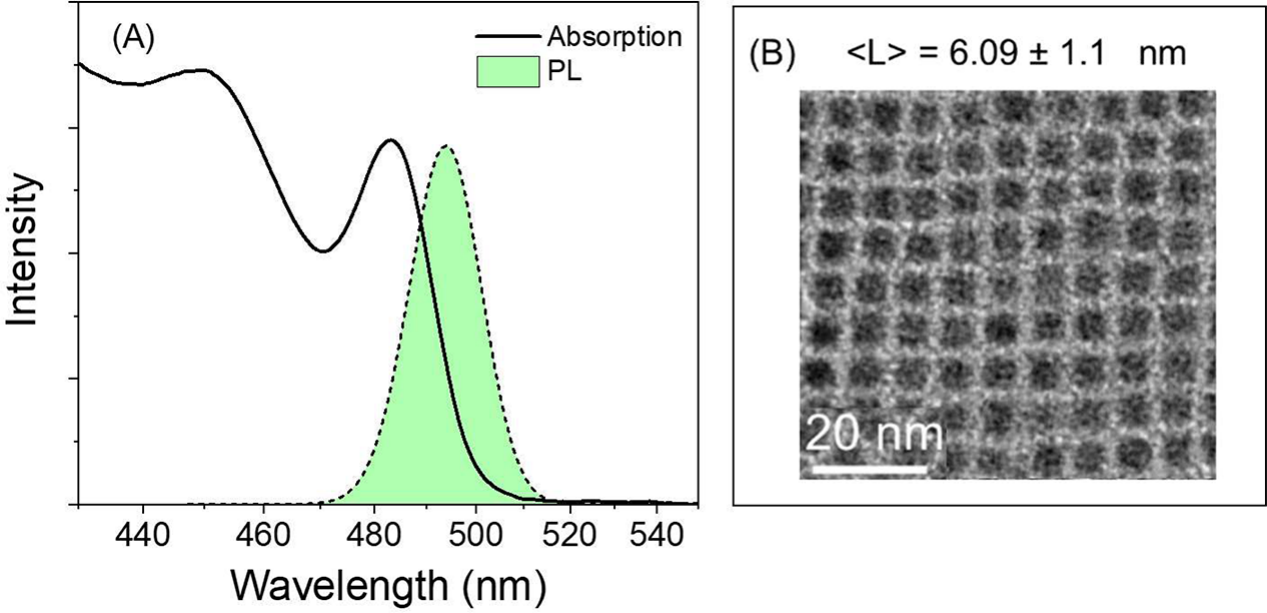
(A) Linear absorption and PL spectra of 6 nm CsPbBr_3_ NCs. (B) TEM image of them with the mentioned average edge-length.

Figures 1 and S2 present the linear absorption and PL spectra of CsPbBr_3_ NC samples studied here, which were determined by TEM imaging to have average edge lengths of the 6.1 and 5.2 nm. The distinct BE exciton transition with observable higher energy bands is indicative of their quantum confined nature. The PL quantum yields (QYs) of both samples are greater than 80% indicating negligible carrier trapping. Results presented here pertain to 6 nm CsPbBr_3_NCs. As shown in the Supporting Information (SI), all findings were verified on 5 nm CsPbBr_3_ NCs as well.

### TRPL

Comparing SX and TRPL experiments on identical NC samples aims to obtain measures of biexciton interaction and emission cross sections without interference from sample specific effects. Furthermore, TRPL simplifies interpretation by avoiding interference of emission and absorption inherent to TA that is at the heart of the SX method. Spectral shifts between biexciton and single exciton emission have been used in the past for estimating the biexciton (XX) interaction energy (Δ_XX_) in various NC samples.^33−35^ Despite the brief lifetime of a biexciton relative to the single exciton state due to Auger recombination, the former component can be isolated even in CW PL at sufficiently high excitation fluences. It is, however, most easily extracted at early delay times using time-resolved emission spectroscopy. In the case of LHP NCs, the latter is advantageous also due to short Auger recombination times relative to those of NCs of other semiconductor materials.

**Figure 2:**
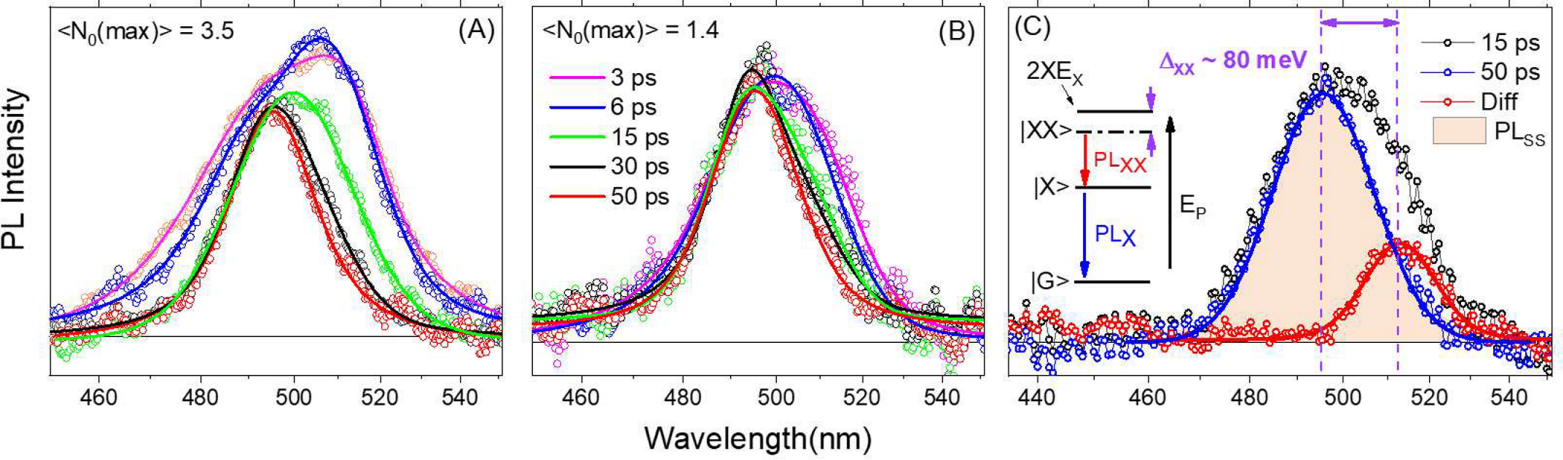
(A) Time resolved PL specta of 6 nm CsPbBr_3_ under high intensity photoexcitation resulting in ⟨N_0_(max)⟩ = 3.5. At early delays broadened PL resulting from high multiexcitons is noted. The broadening at the blue energy side converges to the steady state emission spectrum rapidly leaving a distinct long-lived red shoulder decaying on a longer time scale. (B) TRPL at ⟨N_0_(max)⟩ = 1.4: Lowering in pump intensity eliminates blue extended PL, with a distinct red shoulder which converges on a similar time scale to the steady state emission as in (A), depicted in orange shading. (C) TRPL spectra recorded at 15 ps (black circles) and 50 ps (blue circles) at ⟨N_0_(max)⟩ = 3.5. The latter, and the difference spectrum which is presented in red circles, are both fit to Gaussian functions presented as solid blue and red lines, respectively. The difference of peak positions between red and blue curves estimates a biexciton binding energy of 80 meV. Inset to panel C presents a schematic potential energy diagram clarifying rationale for estimating Δ_XX_.

The homemade Kerr gate PL setup used is depicted schematically in Figure S1, providing a Gaussian IRF of 400 fs fwhm. Figure 2 presents an overlay of TRPL spectra obtained from 6 nm CsPbBr_3_ NCs at various delays following excitation, along with a scaled CW PL spectrum. Panel A is obtained with high intensity pump pulses which provide an average number of absorbed photons per NC (⟨N_0_⟩) of 3.5 within the 1/e intensity diameter. We note that the low signal intensity forces us to collect fluorescence from the entire irradiated volume, thus including a large variation of local excitation densities. At early delays the emission spectrum extends above and below the CW PL. Similar broadening in the emission spectrum has been reported for high multiexciton states in bulk-like LHP NCs as well. As the delay increases, the TRPL spectrum converges with that of CW fluorescence. At the last stages of this convergence, a distinct remnant excess emission band localized on the lower energy side is apparent. At early delay times after ⟨N_0_(max)⟩ = 1.4 excitation, the difference between CW fluorescence and TRPL spectra is concentrated in a well-defined red-shifted band, and converges to the CW PL in accord with the late delay time data in panel A. Clearly the blue-shifted emission emanates from higher multiexcitons which revert through rapid Auger recombination to the biexciton which is more prominent initially after ⟨N_0_(max)⟩ = 1.4 excitation.

The distinct red-shifted residual observed at the final stages of PL convergence to the steady state emission (PL_SS_) is assigned to emission from the relaxed biexciton (PL_XX_). In order to separate this component, TRPL from panel 2(A) collected at 50 ps is subtracted from that at 15 ps. The symmetric residual is well fit to a Gaussian curve (solid red line in panel (C)) and assigned to PL_XX_(λ). As explained in the inset to Figure 1C, the wavelength separation between the centers of PL_XX_ and PL_SS_ reflects an energy separation of 80 meV. Its appearance to the red, and in this case by nearly 20 nm, indicates an intense attractive interaction energy (Δ_XX_) between excitons in these particles as suggested by others.^26^Figure S3 demonstrates the reproducibility of this result in repeated measurements, leading to the relatively narrow uncertainty interval associated with the extracted Δ_XX_.

### SX Experiments

**Scheme 1:**
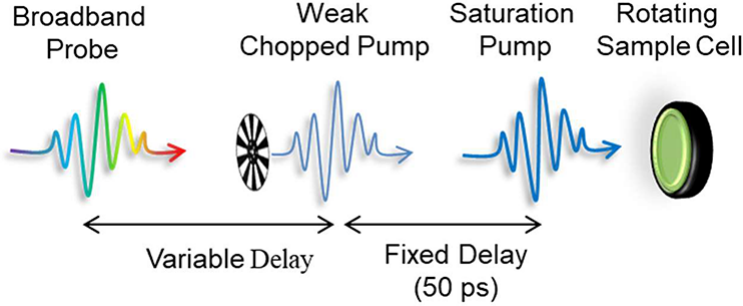
“Spectator Exciton” Experiment A strong above band gap saturation pulse excites the whole sample with at least a single exciton. 50 ps after that, once all the excited particles have relaxed to a single BE exciton, a weak chopped pulse is introduced to further excite the NCs. The time resolved changes are then followed by a variably delayed broadband probe.

**Figure 3:**
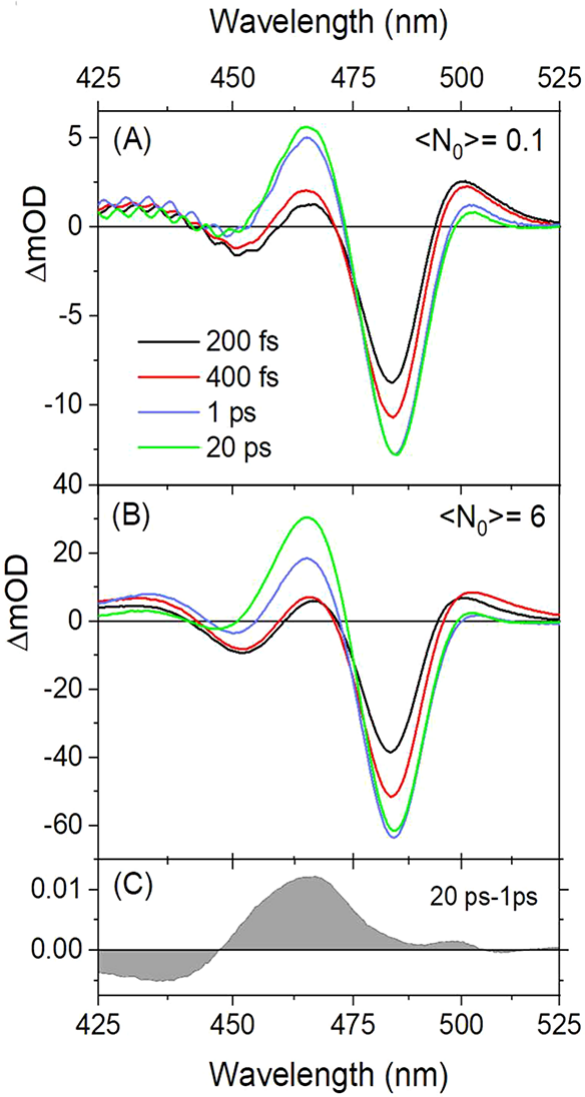
(A) Pump–probe spectra of 6 nm CsPbBr_3_ NCs at different delays after pumping with weak 400 nm laser pulse producing ⟨N_0_⟩ = 0.1. (B) Same as (A) under intense 400 nm photoexcitation leading to ⟨N_0_⟩ = 6. (C) Difference spectrum accumulated over the process of Auger recombination from data in (B) (green minus blue curve).

The principle of the SX experiment is illustrated in Scheme 1. In preparation for the SX investigation, PP experiments were conducted to test consistency with observations in ref (25). The results matched the previous data within error. For weak pump pulses (⟨N_0_⟩ = 0.1) tuned to 400 nm, well above the band gap, TA spectra at a series of delays for the 6 nm NC sample exhibit a prompt subps rise of a sharp BE bleach, together with an equally sharp but weaker induced absorption at shorter wavelengths (Figure 3A). The rise-time of these features has been assigned to hot carrier relaxation to the BE. The TA spectrum obtained after carrier cooling is translated from ΔOD(λ) to a change in cross section [Δσ(λ)] as follows:
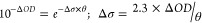
Here θ is the density per unit area of singly excited particles after low fluence excitation calculated from the pump photon density and sample extinction. The result is directly comparable to σ(λ) (Figure S4), and demonstrates that the near-BE bleach induced by a single exciton significantly exceeds 50% of the lowest exciton band. Implications of this in terms of suggested models for the level structure are considered below.

For higher excitation intensity where multiple photons are absorbed per NC with high probability (⟨N_0_⟩ = 6) (Figure 3C), Auger recombination taking place over 10–20 ps is observed (Figure S5). As in ref (25), the difference spectrum over the course of Auger recombination shows that adding a second exciton to the NC, rather than effecting the spectrum near the BE, primarily erases the induced absorption positioned between the lowest two exciton absorption bands (Figure 3B).

**Figure 4:**
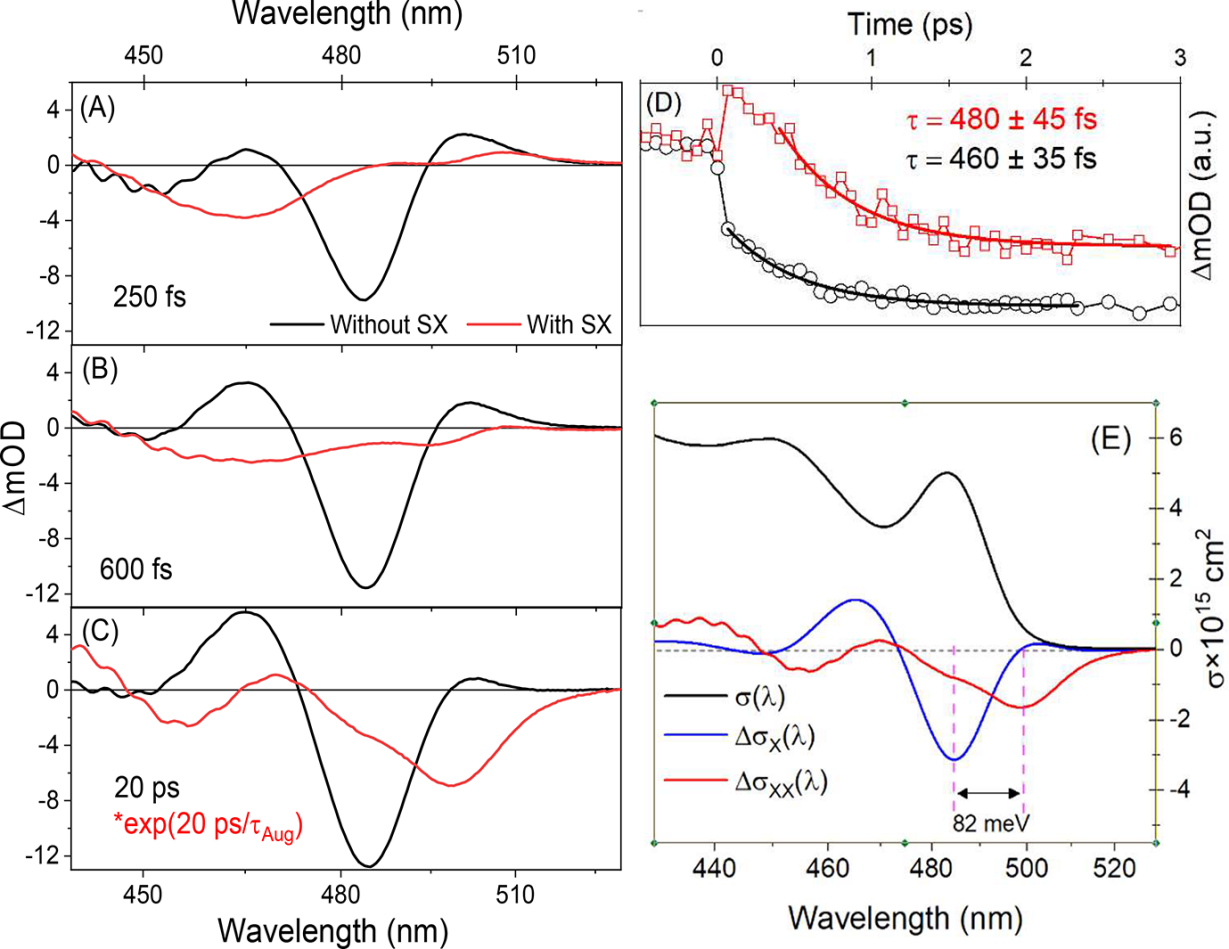
Comparison of TA measurements after weak 400 nm excitation, without (black) and with (Red) spectator excitons at a series of designated pump–probe delays (A–C). Biexciton recombination is compensated for by multiplying it by e^*kτ*^ where *k* is the Auger rate and *τ* the PP delay Panel D presents buildup of BE bleach signal associated with hot carrier relaxation following 400 nm excitation with (λ_Probe_ = 498 nm) and without SX (λ_Probe_ = 484 nm). The exponential fits indicate that carrier cooling is unaffected by SX presence. (E) Comparison of difference cross sections.

In the three pulse SX experiment depicted in Scheme 1, the sample is first saturated with relaxed single excitons. Later it is subjected to a weak pump and probe sequence, which can be compared with results of the same sequence free of SXs. Figure 4 depicts TA spectra with and without SX saturation at different PP delays for weak pump pulses tuned to 400 nm, well above the sample BE. The signatures of carrier cooling, observable over the first ps both with and without the SX saturation, are presented in panel D. This comparison demonstrates that the presence of an SX has no effect on the time scale for relaxation of the nascent carriers to the BE, but affects the ΔOD spectrum drastically.

Conducting conventional 2 pulse PP or SX experiments allows the comparison of spectral changes induced by adding one and then a second excitons in our sample as presented in Figure 4E. Following carrier cooling, presence of a second exciton in the NC induces an additional bleach at the red edge of the difference spectrum, which is roughly twice as broad as that observed for the first excitation. The extent of the red shift is ∼82 meV, which notably matches the biexciton shift obtained from the TRPL data (Figure 2C). We note that the signal due to SX as presented in Figure 4 has been corrected by multiplying it by e^*kτ*^, where *k* is the Auger rate and τ is the delay, to compensate for the rapid ongoing Auger recombination. These spectral changes are presented as Δσ per exciton and are therefore quantitatively comparable. At shorter wavelengths a series of lower intensity alternating positive and negative features appear which are consistent with moderate red shifting in the prominent absorption induced by a single exciton as described above.

**Scheme 2:**
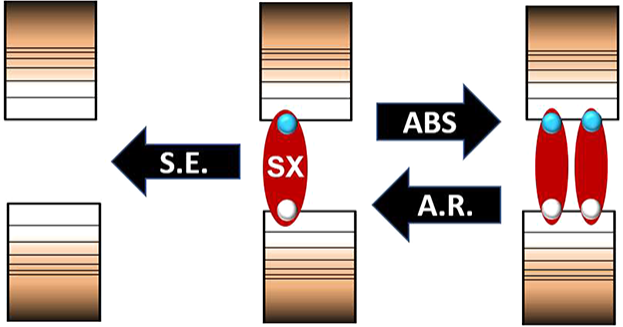
Schematic Illustration of Two Possible Outcomes of Band Edge Irradiation on SX Saturated NCs Photoexcitation results in a biexciton as depicted to the right. This reverts within tens of ps to a single exciton through Auger recombination (A.R.). Alternatively, irradiation stimulates emission (S.E.) to the stable ground state depicted left.

Overlap of induced absorption, absorption bleaching, and SE challenges the interpretation of TA data. In the case of singly excited NCs, the SX approach can separate SE from the other two ΔOD components. In an SX saturated sample, photoexcitation near the BE (unlike that at 400 nm described above) can have two outcomes (Scheme 2). One is absorption and generation of a biexciton, as already discussed. The second, which is specific to near BE excitation, is SE which leads to a ground state NC. In the former case, the biexciton will revert to a singly excited particle within the relatively short biexciton recombination time (here ∼10 ps) leaving no long-lived trace in the difference spectrum. In contrast, SE will transform a single exciton containing an NC to one free of electronic excitation. This will leave a signature opposite in sign and equal in duration to the single exciton ΔOD, typically in the nanosecond time range.

**Figure 5:**
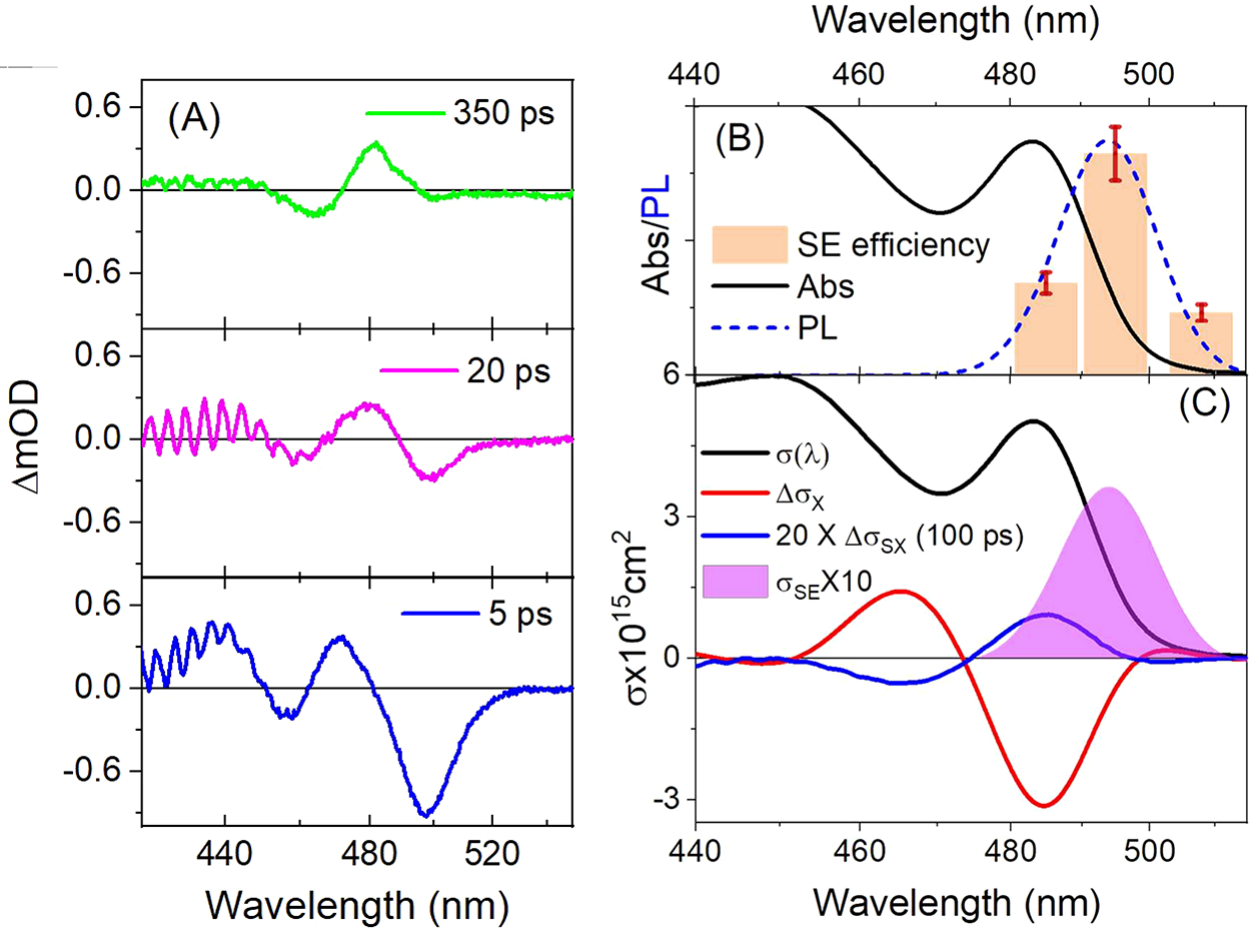
(A) Spectral evolution in an SX experiment with band edge pumping. Diminishing of the red bleach signal though Auger leaves a long-lived residual consisting of an inverted replica of the single exciton TA. (B) Integrated SE yield as obtained from the inverted signal intensity as a function of band edge pump wavelengths (tracking different portions of CW PL; Figure S7). (C) Transient difference absolute cross section spectra compared with the absorption cross section of a single particle (black). The difference cross section per particle without SX at PP delay of 2 ps is shown in red, compared with that obtained with SX at a delay of 100 ps (blue). In Magenta is the stimulated emission cross section (*10) calculated on the basis of these spectra. See SI for details.

To test this, SX experiments were repeated using the same saturation method but shifting the weak delayed pump pulse spectrum to the BE to coincide with the peak of PL. Figure 5 presents the difference spectrum of this sequence at a series of PP delays. TA at 5 ps closely resembles the equivalent SX difference spectrum obtained when pumping at 400 nm, consisting of a broad and red-shifted BE bleach with a band shifting signature at shorter wavelengths. In both cases, the pump must primarily be promoting a second exciton into the SX containing particles and after carrier cooling should produce the same spectral change. However, at 25 ps after Auger recombination has restored all biexcitons to the SX state, a weak inverted replica of the single exciton ΔOD remains, as expected for the presence of SE. This assignment is strengthened by the persistence of this residual out to the latest times probed.

To verify the assignment of the observed residual to SE, SX experiments were repeated for pump pulses tuned throughout the PL spectrum. Results are depicted in Figure 5B, where a coarse stick spectrum presents intensities of the residuals after integrating over the BE absorption lobe at three pump wavelengths, and compared with a scaled PL spectrum of the sample. Agreement within the substantial margin of error confirms the assignment to the action of pump induced emission. In view of the limited spectral width of the latter, we assume henceforth that σ_SE_(λ) tracks the PL spectral distribution precisely (Figure S6). A spectrum of σ_SE_(λ) so derived is portrayed together with the Δσ plot in Figure 5C.

Based on these results, we can determine if 400 nm and BE excitation SX experiments lead to the same ultimate difference spectrum (σ_XX_ – σ_X_ ≡ Δσ_XX_) as they should. The ΔOD spectra after carrier cooling are compared for both experiments in Figure S8 after subtraction of the SE residual and correction for Auger recombination. We find that both experiments present identical below BE induced transparency with weaker alternating shifting features to the blue. Taking into consideration that these spectra have been obtained after subtraction of pump scattering for the BE excitation experiment and are obtained without any normalization, we find the similarity striking, particularly in terms of the most prominent bleach peak near 500 nm. We note that the fast modulations in the blue result from an interference artifact of the continuum with the third harmonic of the NIR generating pulse. Thus, within reasonable margins of error, Δσ_XX_ is obtained with high fidelity from both experiments.

## Discussion

### 1 Biexciton Interaction, and Hot Carrier Cooling

The TRPL results prove consistent with strongly attractive biexciton interaction which in the 6 nm CsPbBr_3_ sample matches Δ_XX_ = 80 meV. This contradicts our previous claim that this interaction is repulsive based on our assignment of the induced TA absorption band to residual BE absorption.^25^ What then can be learned from examining the observations that led to that inaccurate suggestion. As reproduced here and depicted in Figure S9A, the Auger difference spectrum following high intensity above BE excitation differs markedly from that obtained in SX experiments as shown in Figure 5. Adopting the assignment of the induced absorption band to strong red shifting of the second exciton band, biexciton recombination after dense photoexcitation must primarily take place from a “hot” XX state. This is supported by the difference spectrum obtained at the late stages of Auger recombination (3–50 ps) after a moderate excitation intensity (Figure S9B). Since the Auger time scales appear similar in hot or relaxed biexcitons, the reversal of time scales relative to carrier cooling must reflect a slowing down of the latter. Something in the preparation of these ultimately doubly excited states must have extended the duration of hot carrier cooling from the sub ps time scale depicted in Figure 4D, to ∼10 ps. The only variation is that the intense PP experiment produces a distribution with a large average number of excitons absorbed per particle. These recombine rapidly producing doubly excited NCs in a fraction of a picosecond and somehow predisposing the hot biexcitons to much slower cooling. It is important to point out that this situation is very different than that portrayed in Figure 4D where we compare cooling rates of a single hot exciton in an otherwise cold NC, with that of an equally hot exciton introduced into a equally cold SX containing particle, demonstrated to have identical cooling times.

While identifying the mechanism behind this slowing will require further investigation, the most obvious candidate would be the heating of the optic phonon modes by a sequential cascade of Auger recombinations. Such a slowing of carriers following dense photoexcitation of bulk semiconductors is well-known and coined the “Hot Phonon” effect, including in bulk LHP.^36−38^ An analogous effect of hot phonons in the context of semiconductor NCs has also been reported.^39−41^ While a recent study reports a slow component to carrier cooling in LHP NCs after intense multiphoton absorption, the average lifetimes are far lower than observed here.^42^ Efforts are ongoing in our laboratory to clarify the impact of phonon temperatures on hot carrier cooling in QC LHP NCs by complementary methods. It is noteworthy that no signs of spin blockades to the relaxation of hot biexcitons were observed in these SX experiments akin to those detected in similar experiments on CdSe NCs,^29^ perhaps reflecting heavy atom induced rapid spin flipping in LHPs.

### 2 Stimulated Emission Cross section (σ_SE_): Determination and Significance

**Figure 6:**
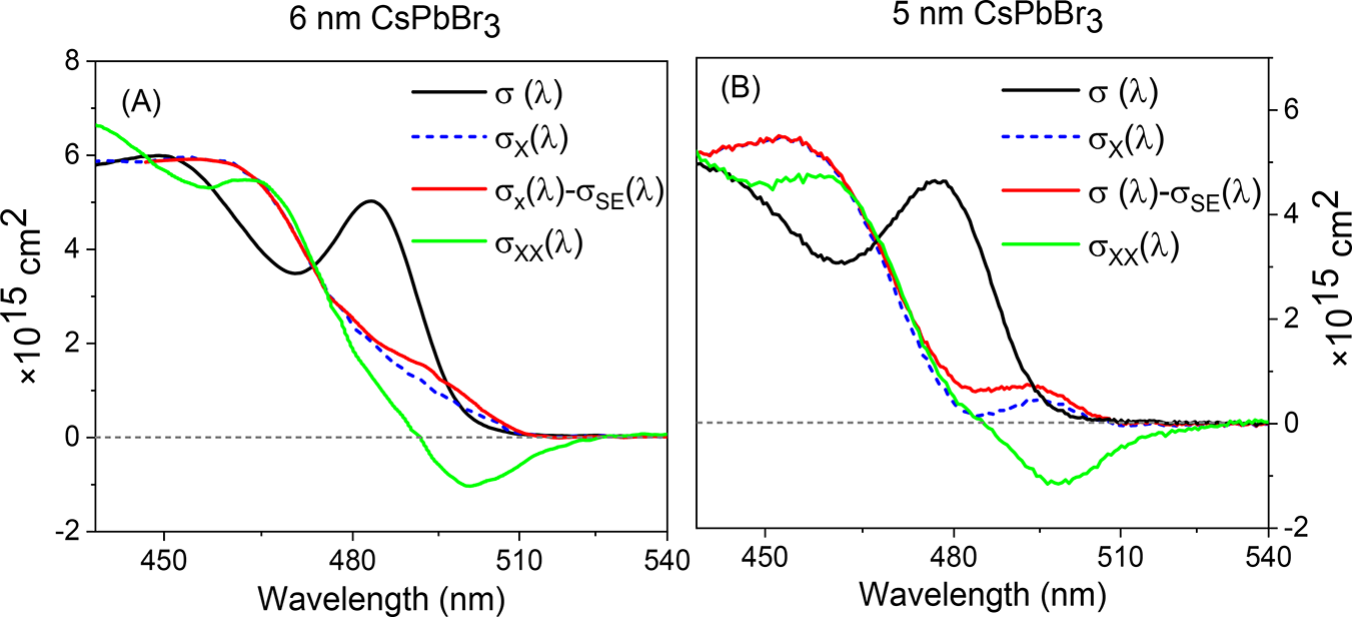
Comparison of the apparent absorption cross sections of a ground state NC, one containing one, and one containing two relaxed excitons, for (A) 6 nm CsPbBr_3_ and (B) 5 nm CsPbBr_3_. In both cases, no net gain is observed at any wavelength under single excitation of the NCs, while a large gain is obtained in doubly excited case. The purely absorptive cross-section is depicted as well, clearing of the effects of stimulated emission obtained by the SX method.

After resolving the TA difference spectra and SE contributions thereof in terms of absolute cross section changes, we are able to compare the apparent cross sections of both with that of the ground state. Using the single exciton Δσ, and σ_SE_, the purely absorptive contribution to σ_X_, σ_X_(AB) can be calculated as σ_X_(AB) ≡ σ_X_ – σ_SE_. Furthermore, σ_XX_ can be obtained by adding the biexciton difference spectrum to σ_X_. All of these spectra are summarized in Figure 6 and prove that single excitation in these NCs does not produce net optical gain at any wavelength covered by our experiments. Furthermore, the peak amplitude of σ_SE_ is roughly 6.7 times smaller than that of the single exciton bleach band in Δσ. In contrast, a relaxed biexciton does exhibit a significant region of enhanced transmission below the BE in Δσ_XX_. Since both absorption bleach and SE lead to negative ΔOD amplitudes, this feature must be assigned to SE since no bleachable absorption preceded it. Identical results were observed in an analogous sample of 5 nm sided CsPbBr_3_ NCs and are depicted in panel B, demonstrating generality in quantum confined particles of this material.

Contrary to a recent report where even a single polarized exciton can generate net optical gain,^27^ the spectra presented above in Figure 6 show this is not the case once the coherent charge pair is dephased. Using the SX approach, the cross section of single relaxed exciton stimulated emring presence of excited state absorption (Figure S6).

The amplitudes of Δσ_X_ and σ_SE_ must now be rationalized versus predictions of electronic structure models. Relative to the BE exciton absorption in σ(λ), they must reflect state degeneracies and transition selection rules between the discrete low energy electronic levels. Quantitative comparison is however complicated in these samples by several factors. If one accepts that the induced absorption peaking at 460 nm in Δσ_X_ in Figure 4E is actually a shift to a lower energy of the second exciton band in σ(λ) at 445 nm, it may overlap and partially cancel the bleach due to BE state filling. In NCs of typical semiconducting materials, biexciton interactions and electron–phonon shifts are much smaller than typical exciton band widths, allowing the relative transition intensities to be evaluated be changes in peak heights in Δσ and σ(λ). Here, in view of significant excited state absorption peak shifts, spectral fitting and band area evaluations are unavoidable. Even then mutual band cancellation will lead to model dependence in fitting.^21^

Another challenge involves uncertainty of the buildup of quasicontinuous absorption as we shift the wavelength to the blue. At a sufficiently short wavelength, NC absorption closely resembles that of the bulk and is proportional to the particle volume. The transition between this and the discrete BE levels is not sudden and challenging to model. It is however a necessary component in band fitting of the spectra, introducing additional uncertainty. In a recent study, Barfüßer et al. showed that this separation can be aided by cryogenic cooling spherical CsPbBr_3_ particles.^21^ This approach might aid in resolving this issue, but conducting an SX experiment under cryogenic cooling will be challenging.

We accordingly settle for a semiquantitative comparison of the BE features in σ(λ) and Δσ(λ) in Figure 5. Continuum contributions as well as overlap with higher exciton transitions for the former, along with band cancellations discussed for the latter, make the observed areas of BE absorption peak in σ(λ) and the bleach in Δσ(λ) upper and lower limits thereof, respectively. Even so bleach maximum at 485 nm in Δσ(λ) is nearly 70% as large as that of the BE absorption in Δσ(λ). In view of the similar band widths, and since the former is a lower and the latter an upper limit of the real amplitudes, the corrected ratio of single exciton bleach to the BE exciton absorption must be close to unity. This is in contrast to expectations based on 3 Cartesian bright components which predict single exciton state filling blocks only one-third of the BE absorption contrary to our observations.^43,44^ Another model used for the analysis of polarization dependent TA by Liu et al. would predict state filling to block 50% of the band BE exciton absorption,^27^ much closer to our observation. But would it correctly predict the intensity of σ_SE_?

Turning to the inclusion SE effects, there is another absolute signal ratio that should be predicted from the model of electronic structure: Δσ/σ_SE_. Assuming strong spin orbit coupling renders the spin a poor quantum number, one might predict an SE component which is equal to the bleach while exciton coherence persists, and a decay to 1/3 after exciton dephasing. The former equality would also be predicted for the level scheme described by Liu et.al. for the coherent exciton, and a BE state filling effect of 50% which is closer to our observation.^27^ In contrast, it would also predict a relaxed stimulated emission cross section close in intensity to the bleach, even farther from the measurement. In reality we observe an intensity ratio of 6 ± 1 (Figure S6). If either electronic structure model holds, the only way to reconciliate the observed ratio with the low intensity of σ_SE_ is by invoking the presence of a significantly stabilized dark exciton level competing for population with the bright triplet. Even so the suggested separations between these states would not suffice to darken a thermal room temperature mixture of these levels sufficiently to produce such a large ratio.^43,23^ Accordingly none of the currently suggested models of CsPbBr3 NCs level structure comes close to agreement with both amplitudes of single exciton bleach and of SE cross sections.

### 3 Δσ_X_ vs Δσ_XX_

We now consider differences between one- and two-exciton cross section difference spectra. These are the TA spectra of a pristine sample and one obtained from SX containing particles, resolved into absolute cross section changes. In previous SX experiments on CdSe and PbS NCs,^29,45^ a comparison of Δσ_X_ and Δσ_XX_ revealed relatively similar spectra for both, indicating moderate biexciton shifts and negligible electron–phonon coupling, and a linearity of integrated bleaching bands at the BE with each exciton. Furthermore, in both cases, no contributions of stimulated emission to Δσ_X_ were isolated. In contrast, here we uncover measurable SE in particles known to exhibit stronger shifts due to biexciton interactions and electron–phonon coupling as demonstrated by the TRPL data.^27,46^

As depicted in Figure 4E the bleach peak in Δσ_XX_, while equal in area with that in Δσ_X_, is strongly red-shifted and broadened by a factor of 2. Instead of an induced absorption band in the blue, Δσ_XX_ contains a zero-area peak shifting feature, which is consistent with a mild additional peak shift of the induced absorption. To explain the extreme broadening and red shifting of the BE bleach in Δσ_XX_, both SE and state filling contributions need to be considered. The second exciton might be expected to induce a bleach equal to that of the first. However, the contribution of double occupancy should boost the effect of SE from the relaxed and incoherent excited state. In the case of Δσ_XX_(λ) population of e/h states which can emit is much larger and this should greatly enhance the ratio between the state filling vs SE contributions. On the one hand this would explain the strong red shifting of the apparent bleach lobe in Δσ_XX_(λ), in view of the strong Stokes shift of the biexciton PL. It would not explain the similar areas for the bleach in Δσ_XX_(λ) and Δσ_X_(λ) as the former will have enhanced SE. Regrettably, the SX method can measure only σ_SE_ of the single exciton state. Nonetheless, we can predict enhanced impact of SE on Δσ_XX_ with confidence. Making this distinction is important in the case of LHP NCs. Before considering SE, the BE bleach in Δσ_*x*_(λ) reflects a subtraction of the initial absorption in the ground state, superimposed by the excited state absorption of singly excited NCs. The latter being red-shifted by Δ_XX_ leads to a blue shifting bleach peak, with occasional appearance of shallow induced absorption on the red edge depending on the band widths and amplitude of Δ_XX_. While SE contributes with the same sign as the bleach (negative ΔOD), it is red-shifted both by Δ_XX_ and by Stokes shifting due to lattice relaxation.^47,48^ In the case of Δσ_*x*_(λ) SE is weak and therefore of little consequence, but not for Δσ_XX_(λ). To test this the bleach band in Δσ_XX_(λ) is compared with biexciton emission detected in TRPL (Figure S10). The match is less than perfect, with the bleach peak in Δσ_XX_(λ) falling somewhere between those of PL_X_ and PL_XX_. This might indicate an imperfect extraction of the latter spectrum by the fitting depicted in Figure 2C. This does not contradict the assignment of the red-shifted enhanced transmission in Δσ_XX_(λ) primarily to SE from the doubly excited state.

## Conclusion

We have utilized a 3-pulse ultrafast spectroscopic method coined the “Spectator Exciton” (SX) approach to capture the stimulated emission cross section in singly excited quantum confined NCs, even when masked by overlapping excited state absorption and ground state bleach. Difficulty in obtaining this parameter has been a long-standing obstacle in the way of band edge state degeneracy and transition intensity determination in NCs of many semiconducting materials. Here we applied this method to CsPbBr_3_, a family of NCs whose BE electronic structure is the subject of an active ongoing debate. Our results show that, in 5–6 nm CsPbBr_3_ NCs, a single exciton bleaches more than half of the intense BE absorption band, and the cross section for stimulated emission from the same state is nearly 6 times weaker. Comparing these findings with predictions of recent electronic structure models for this material proves their shortcomings in explaining both measures, proving the importance of this new input in resolving this debate and the need for further study. Finally, bolstered by femtosecond time-resolved PL measurements on the same sample, the SX results verify that biexciton interaction is intensely attractive with a magnitude of ∼80 meV. In light of this observation, a previous suggestion that biexciton interaction is repulsive is reassigned to hot phonon induced slowdown of carrier relaxation leading to direct Auger recombination from an excited state.

## Supporting Information Available

Schematic layout of the TRPL setup and IRF; Absorption, Steady state PL, and TEM image of 5 nm CsPbBr_3_ NCs; Different sets ΔXX estimate from TRPL; Estimation of absorption cross section and bleach per exciton for 6 nm CsPbBr_3_; Auger kinetics, Relative intensity comparison between the σ_*x*_(λ) and σ_SE_(λ); Band edge pump tunability for determining σ_SE_(λ); Difference spectrum under different measurement conditions; Time-resolved data for 5 nm CsPbBr_3_ NCs; Procedure to calculate the SE efficiency and σ_SE_(λ). (PDF)
