# Supplementary material for: Spectator Exciton Effects in Nanocrystals III: Unveiling the Stimulated Emission Cross Section in Quantum Confined CsPbBr3 Nanocrystals
Source: J Am Chem Soc. 2024 Jul 15;146(29):20241–50. doi: 10.1021/jacs.4c05412 (PMC11273341; doi:10.1021/jacs.4c05412)
Supplement: Supplementary file 1 — ja4c05412_si_001.pdf [file ja4c05412_si_001.pdf]

Supporting Information

Spectator exciton effects in nanocrystals III: Unveiling the stimulated emission cross section in quantum confined $CsPbBr_3$ nanocrystals

Apurba De[‡,†], Soumyadip Bhunia[‡], Yichao Cai[‡,⊥], Tal Binyamin[‡], Lioz Etgar[‡] and Sanford Ruhman[‡]*

[‡]*Institute of Chemistry, The Hebrew University of Jerusalem, Jerusalem-91904, Israel*

**Corresponding author: sandy@mail.huji.ac.il*

(A)

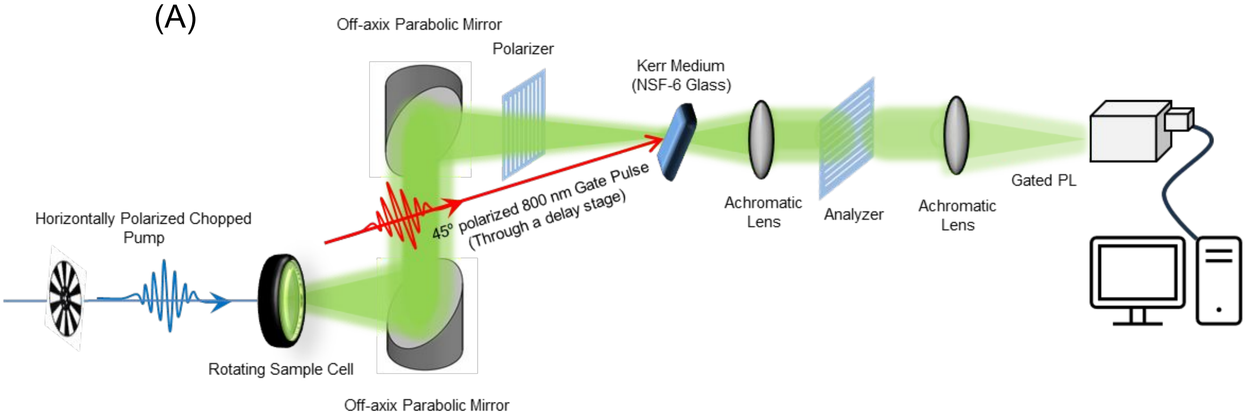

(B)

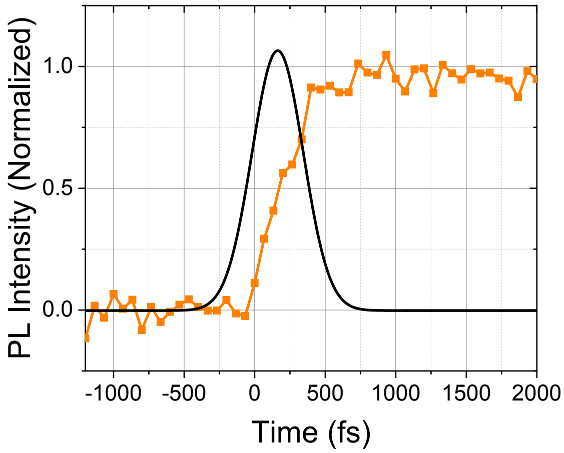

Figure S1. (A) The qualitative layout of our home-built time-resolved PL setup (Optical elements drawn are not scaled). Details of the setup are provided in the experimental section. (B) Growth kinetics of the DCM dye (dissolved in the acetonitrile solvent) fluorescence with excitation at 400 nm. The PL intensity was integrated over wavelengths of 550-750 nm. The Gaussian fit to the first derivative of the PL rise is also presented in the black curve. The FWHM of this Gaussian curve, corresponding to the instrument response time, is approximately 400 fs.

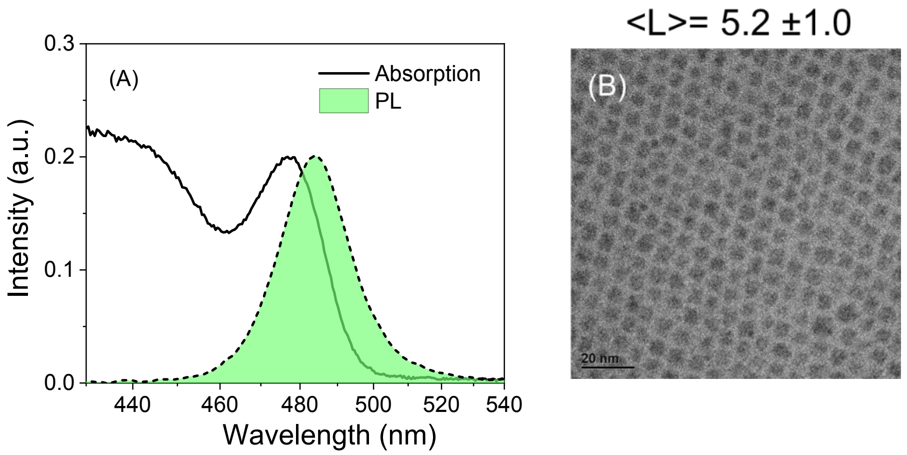

Figure S2. (A) Linear absorption and PL spectra of 5 nm $CsPbBr_3$ NCs. (B) TEM image of them with the mentioned average edge length. The scale bar in the TEM image is 20 nm.

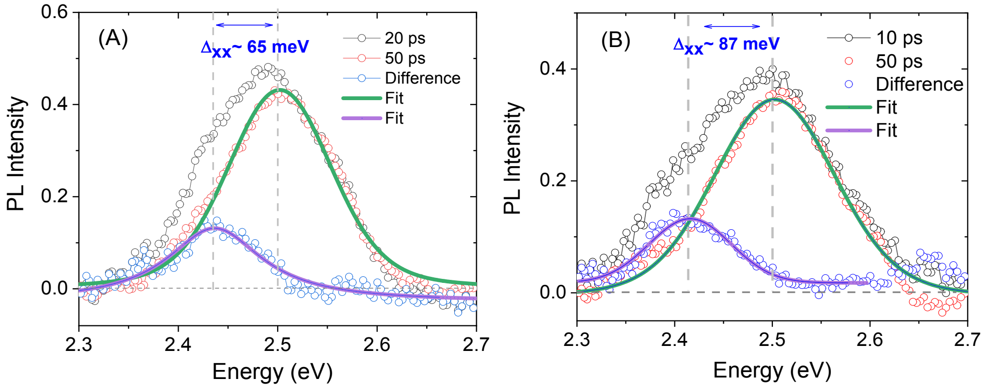

Figure S3. TRPL spectra of 6 nm $CsPbBr_3$ NCs for subsequent measurements showing the uncertainty associated with the extracted ΔXX.

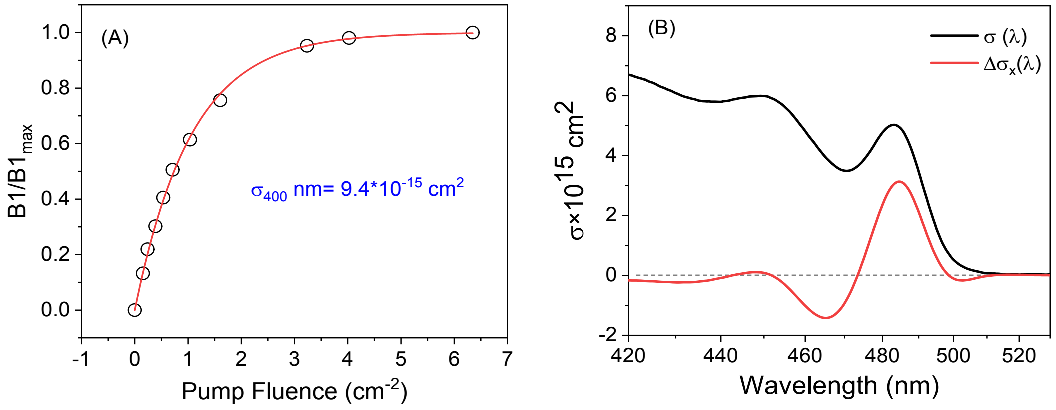

Figure S4.(A) Determination of absorption cross-section of the NCs by bleach saturation experiment. The method is similar as described in the previous report.[1] (B) Comparison of absorption cross section with the bleach difference cross section per exciton for 6 nm $CsPbBr_3$.

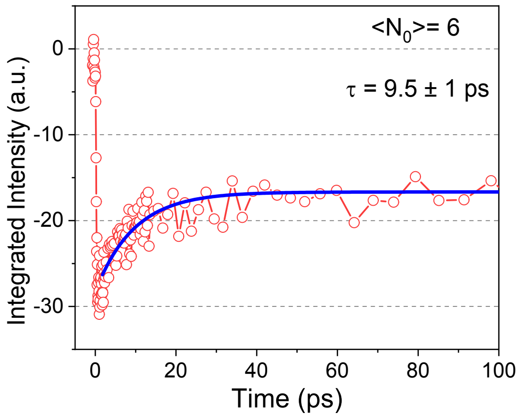

Figure S5. Auger kinetics as obtained for Figure 3B from the band integral calculated as dipole strengths $\Delta D = \int d\nu \frac{\Delta OD}{d\nu}$, over the range 450-530 nm.

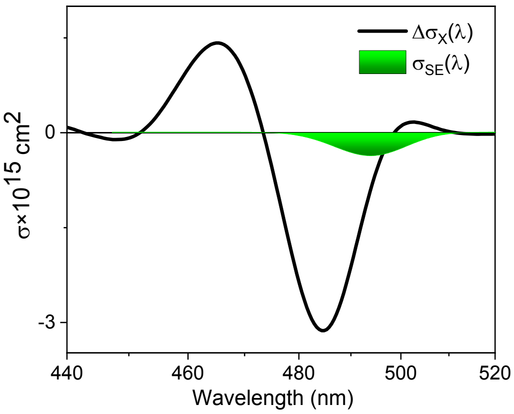

Figure S6: Comparison between the relative intensity of the single exciton cross-section change vs stimulated emission cross-section as estimated from the SX experiments.

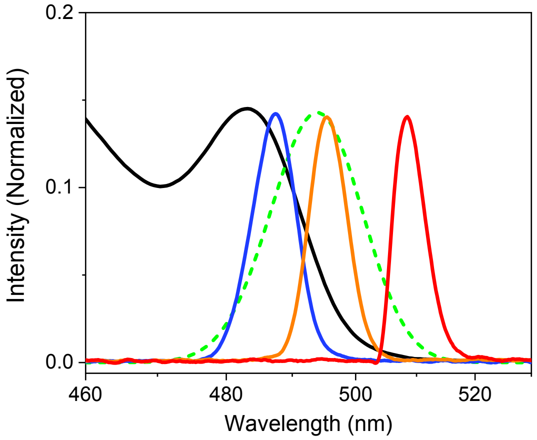

Figure S7: Tuned pump wavelength utilized in band edge SX experiments, tracking different portion of the CW PL.

SE cross section σ_{SE} determination:

The probability for stimulating emission at low pump intensity is $P_{SE} = J_p \times \sigma_{SE}$, where J_p is the measured pump photon flux. We remind the reader that the small negative residual SX signal directly quantifies those spectator containing particles which undergo SE. P_{SE} can thus be directly calculated from the ratio between that long delay spectator exciton residual (remaining after Auger recombination), to the initial ΔOD following SX saturation: $P_{SE} = -\Delta\sigma_{SX}$ (100 ps) / $\Delta\sigma_X = -\Delta OD_{SX}$ (100 ps) / ΔOD_X. In the SX experiment pumping at 495 nm, $-\sigma_{SX}$(100 ps, 450 nm) /$\Delta\sigma_X$ = -2.2 /94 = 0.023. Using the pump photon fluence this leads to σ_{SE} (495 nm) = 3.6E-16. After demonstrating the coarse-grained match between wavelength dependence of SE to that of PLE, we have assumed an identical spectrum for $\sigma_{SE}(\lambda)$.

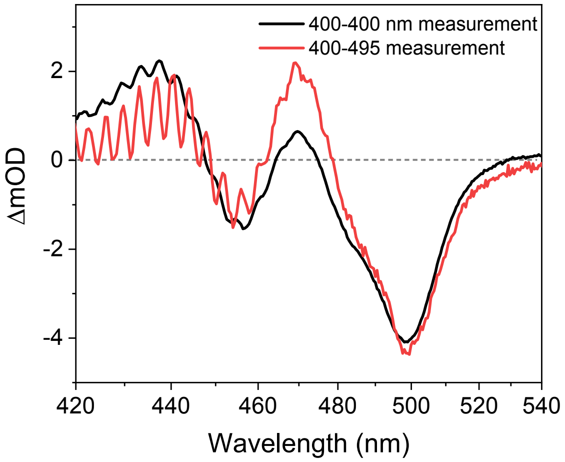

Figure S8. Difference spectra obtained from two different SX experiments. In the 400-400 nm case, the spectrum is Auger adjusted to avoid any signal loss due to biexciton recombination. While in 400-495 case it is both Auger and stimulated emission adjusted to represent the true picture.

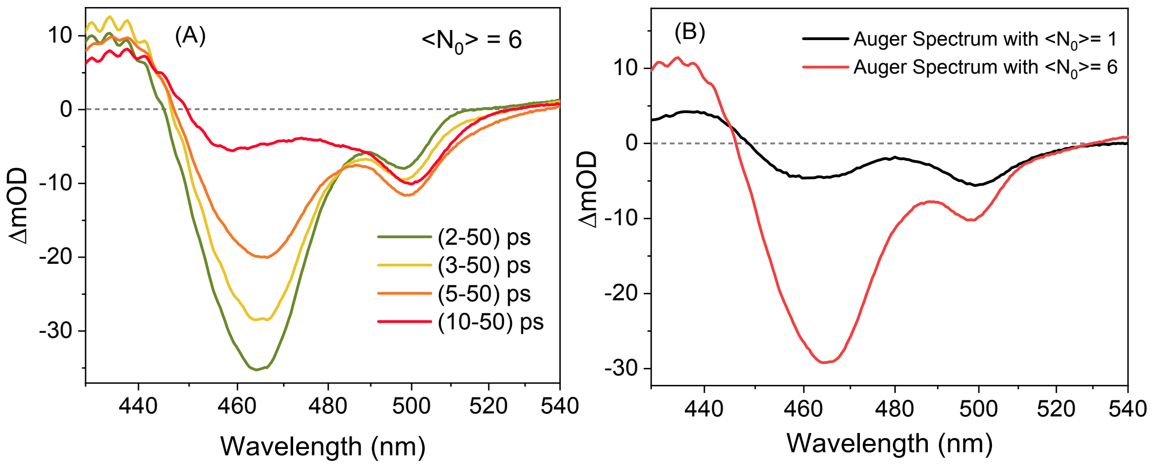

Figure S9. (A) Time evolution of Auger spectrum under above band gap intense photoexcitation. The Auger spectrum at early time looks significantly different than that obtained from SX experiments (Figure 3A a7 S4). This is because biexciton recombination under dense photoexcitation must be taking place from 'hot' XX states, thus appearing dominantly to the blue side of the bleach. (B) Effect on Auger spectrum under two photoexcitation conditions. Lowering in intensity readily leads to significantly reduced 'hot' XX interaction.

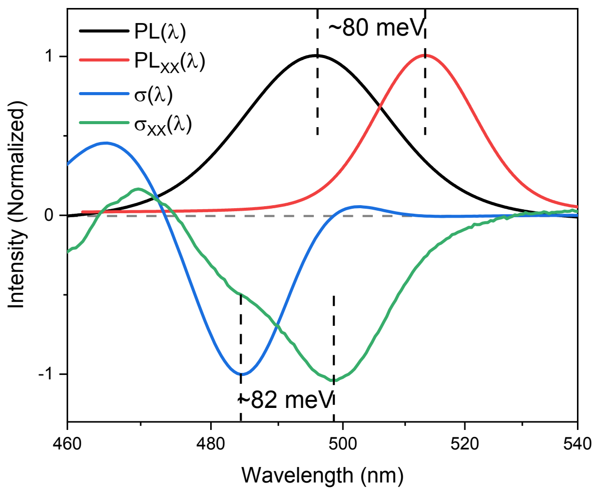

Figure S10. Normalized plot of the data obtained from figures 4E and 2C to compare the time resolved absorption and PL data.

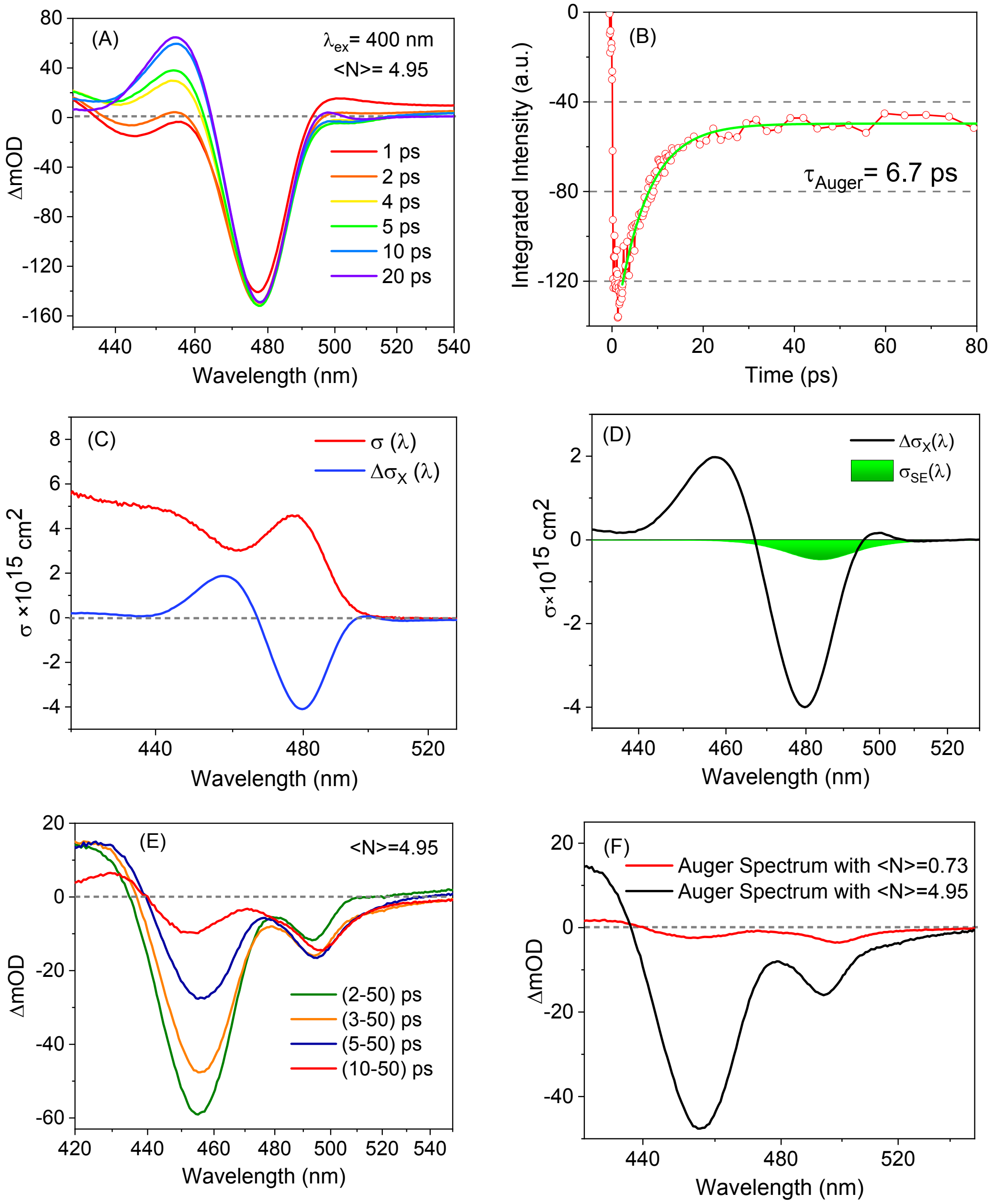

Figure S11: All data presented here are for 5 nm $CsPbBr_3$. (A) Pump-probe spectrum of NCs under intense 400 nm photoexcitation during the Auger process. Note that similar to 6 nm, there is almost no activity in the bleach position during the process while most of the changes are in the induced absorption in the blue to the bleach. (B) Auger kinetics as obtained for figure SA from the band integral calculated as dipole strengths $\Delta D = \int dv \frac{\Delta OD}{dv}$, over the range 430-530 nm. (C)) Comparison of absorption cross section with the bleach difference cross section per exciton. (D) Comparison between the relative intensity of the single exciton cross-section change vs stimulated emission cross section as estimated from the SX experiments. (E) Time evolution of Auger spectrum under intense above band gap photoexcitation. (F) Effect on Auger spectrum under two photoexcitation conditions.

REFERENCES

(1) Dana, J.; Binyamin, T.; Etgar, L.; Ruhman, S. Unusually Strong Biexciton Repulsion Detected in Quantum Confined CsPbBr3 Nanocrystals with Two and Three Pulse Femtosecond Spectroscopy. *ACS Nano* **2021**, *15* (5), 9039-9047. DOI: 10.1021/acsnano.1c02123.
